# Climate, race, and the cost of capital in the municipal bond market

**DOI:** 10.1371/journal.pone.0288979

**Published:** 2023-08-09

**Authors:** Erika Smull, Evan Kodra, Adam Stern, Andrew Teras, Michael Bonanno, Martin Doyle

**Affiliations:** 1 Nicholas Institute for Energy, Environment & Sustainability, Duke University, Durham, NC, United States of America; 2 Breckinridge Capital Advisors, Boston, MA, United States of America; 3 Civil and Environmental Engineering, Northeastern University, Boston, MA, United States of America; 4 Nicholas Institute for Environmental Policy Decisions, Duke University, Durham, NC, United States of America; Massey University - Albany Campus: Massey University - Auckland Campus, NEW ZEALAND

## Abstract

Both climate risk and race are factors that may affect municipal bond yields, yet each has received relatively limited empirical research attention. We analyzed > 712,000 municipal bonds representing nearly 2 trillion USD in par outstanding, focusing on credit spread or the difference between a debt issuer’s interest cost to borrow and a benchmark “risk-free” municipal rate. The relationship between credit spread and physical climate risk is significant and slightly positive, yet the coefficient indicates no meaningful spread penalty for increased physical climate risk. We also find that racial composition (the percent of a community that is Black) explains a statistically significant and meaningful portion of municipal credit spreads, even after controlling for a variety of variables in domains such as geographic location of issuer, bond structure (e.g., bond maturity), credit rating, and non-race economic variables (e.g., per capita income). Assuming 4 trillion USD in annual outstanding par across the entire municipal market, and weighting each issuer by its percent Black, an estimated 19 basis point (bp) penalty for Black Americans sums to approximately 900 million USD annually in aggregate. Our combined findings indicate a systemic mispricing of risk in the municipal bond market, where race impacts the cost of capital, and climate does not.

## Introduction

### Context

The nearly 4 trillion USD municipal bond market provides capital to states and local governments, water and electric utilities, school districts, and myriad special districts, primarily to provide essential infrastructure and services to community residents across the United States. Investors in municipal bonds are repaid from taxes or fees collected within the service area that issues the municipal bond. Each service area has unique tax and/or rate bases with specific governance and demographic histories and characteristics, and each service area is exposed to distinct risks, whether climate/environmental, economic, or demographic. Credit quality in the municipal bond market is thus inseparable from the particular characteristics of the service area population and its geography, and investors’ perceptions of those risks influence bond prices.

Climate risk has not traditionally been assessed in municipal credit analysis. When considered, climate risk assessment is increasingly conducted as part of professional investors’ Environmental, Social & Governance (ESG) analyses. To inform their decisions, professional investors are purchasing bespoke climate data sets to help them price such risk [1]. In contrast, while race was historically considered explicitly in municipal investments [2], current market participants are presumed to be agnostic with regard to risk associated with race. Separate from integrated ESG analyses, race may be evaluated for impact investments, via socially responsible (or “thematic” / “values-driven”) investing to direct capital to issuers based on specific restorative justice and social equity factors. For risk and municipal credit fundamentals, however, race is not supposed to be a direct consideration.

Despite the clear importance of both climate and race across municipal finances, there has been limited study of the relationship between each factor on bond prices and credit spreads, i.e., the difference between a debt issuers’ interest cost to borrow and a benchmark “risk-free” municipal rate. Climate risk and municipal credit spreads have received recent academic attention, albeit primarily focused on specific climate-related topics. For example, Goldsmith-Pinkham et al. 2019 and Painter 2020 evaluated the impact of sea level rise on municipal bond prices, thus constrained to municipal debt related to very specific geographies [3, 4]. Auh et al. 2022 tested the effect of historical natural disasters on municipal bond spreads [5], examining the market’s reaction to specific events but not necessarily long-term risk expectation. With regard to race, race has not been directly investigated with respect to municipal bond price or yield influence for the entire market. For instance, Bruno and Henisz 2022 and Dougal et al. 2019 study how racial justice factors influence bond prices and how investors respond to Historically Black College and University (HBCU) bonds, respectively [6, 7], while Bergstresser et al. 2013 looks at race as a component of community fractionalization [8]. None of these previous studies examine issuer-level racial demographics specifically. A recent working paper by Eldemire-Poindexter et al. 2021 examines a Black Tax using racial composition as a variable, and is thus the most comparable study, yet their dataset focuses exclusively on city and county municipal bonds and uses county-level racial demographics [9].

This study explores two main issues. First, we ask whether physical climate risk is priced into bond yields. Environmental considerations and climate risk have grown in prominence and are now more accepted in the investment community as both present and future risks [10, 11], even if the price impacts are less straightforward. The municipal bond market, in particular, is considered by many to be on the “front lines” of the climate crisis, since cities cannot relocate due to adverse climate change impacts (compared to companies) [4], because residential properties that generate tax revenues for bond repayment are underinsured from physical climate hazards [12], and because local infrastructure requires significant and ongoing planning, governance and investment to maintain climate resiliency [13]. The surprisingly limited empirical research on climate risk and municipal bond yields has shown some price impacts, where increased climate risk results in higher yields, especially for longer duration bonds [3, 4]. Moreover, documented increases in extreme weather events, mega-droughts, and sea-level rise have elevated salience of climate risk in the financial sector [10, 11]. However, given the potential impact of climate change and climate-related events on municipal bonds (e.g., wildfires, floods, sea level rise), the overall dearth of empirical work at the interface of climate risk and municipal bond market is striking.

Second, we ask whether Black Americans effectively pay more for municipal debt, after controlling for economic conditions of debt-issuing communities. We focus on Black communities because they have been subjected to racial and economic resource segregation and persistent negative income and wealth impacts for multiple generations (e.g., redlining practices in many U.S. cities leading to punitive borrowing rates) [2, 14]. To our knowledge, this is the largest-scale study to date analyzing the relationship among granular, issuer-specific climate and race data and the cost of capital in the municipal bond market. Our combined findings indicate a systemic mispricing of risk in the municipal bond market, where communities with greater percentages of Black residents pay more for municipal debt, but communities with higher climate risk do not.

## Conceptual approach

Many cities and local governments who access the municipal bond market issue bond series in various years, each with multiple and varying maturities. Therefore, if climate risks are salient for bond pricing, investors would consider potential climate trajectories and price bonds according to the expected risk in the years leading up to maturity and/or the year of maturity. The further out in the future, the more uncertainty there is in climate change models and impacts. It is therefore plausible that investors would demand a higher yield from issuers (e.g., cities) with higher climate risk and would demand a higher yield for bonds maturing in later years, to compensate for additional risk and greater uncertainty. With regard to race, if municipal bond investors hold implicit or explicit negative biases about Black communities, then the market would show a yield penalty for communities with a higher percent of Black residents, even after accounting for non-race-based economic or demographic variables.

We seek to empirically test whether physical climate risk and racial bias are priced in the municipal bond market through a comprehensive analysis of yields on all municipal bonds outstanding as of end of April 2022. To our knowledge, this is the most extensive dataset used in a study to investigate how issuer-level climate or race relate to yield in the municipal bond market. Municipal bonds provide a unique lens to explore racial bias and physical climate risk because of the place-based nature of the market [3]. Municipal bond investors directly invest in communities with specific climate and demographic/racial characteristics, and communities use investments to manage economic needs and, increasingly, to finance climate resilience and adaptation. Additionally, investors in municipal bonds are primarily concerned with downside risk and less so upside potential; bond investors receive cash flows subject to the ability of issuers to pay annual interest payments and repay principal, as opposed to increased bond valuations [3]. Prices and yields tend to reflect the likelihood of full repayment, and changes in prices and yields reflect the impact of any risk factor that could limit full repayment.

We sample a subset of Bloomberg’s outstanding municipal bond universe comprising non-sinkable bonds that are not sold via private placements to assess whether the municipal market is sensitive to race and climate variables. We analyze data for individual Committee on Uniform Securities Identification Procedure (CUSIP) numbers, which are 9-digit unique alpha-numeric identifiers for all registered stocks and bonds in North America. We look at both the entire municipal bond market, along with a subset of the bond market made up of only water and sewer revenue bonds. For climate risk, water and sewer bonds only are analyzed because water and sewer utilities have a clear exposure to physical climate risks (e.g., reliance on sufficient water supply) while non-environmental type credit issuers (e.g., school districts) would not. For racial bias, water and sewer bonds are a relevant subset because actual default risk is near zero: only 2 out of 113 total municipal bond defaults from 1970–2019 have been in the water/sewer sector [15], and high customer delinquency rates are rare (i.e., people pay their water bills, with delinquency rates often <1%).

We test our hypotheses via a hedonic pricing analysis where we account for bond structure, rating, state of issue, climate and race, and other economic variables. We test the following hypotheses:

- municipal bonds issued by communities with greater physical climate risk do not pay higher yields, but water/sewer bonds issued by those same communities do pay higher yields (receive lower prices) [3, 4],
- municipal bonds issued by communities with a greater percentage of Black individuals pay higher yields (receive lower prices) on their municipal bonds, and this is true for all municipal bonds and for water/sewer bonds only [7, 9].

## Methods

### Municipal market data

We employ national-scale statistical modeling on a subset of the entire municipal bond market as of April 27, 2022. For each CUSIP within our dataset, we combine yield and spread with bond structure data (e.g., coupon), bond rating from Standard and Poor’s (S&P) and Moody’s, issuer socioeconomic and demographic data (e.g., percent of community that is Black, median household income), and climate risk data tied to the maturity year of the bond. We implement the analysis with spread at issue as the response (dependent) variable, and with market spread as of 4/27/2022 as the response variable. For spread at issue, we also include data on the market conditions, since buyer and seller activity and advantage can vary significantly on different issue dates, even if within the same week. For both response variables, we run the models for the whole municipal bond market and water and sewer revenue bonds only.

For the market spread analysis, datapoints for the whole municipal market were pulled from the entirety of municipal market outstanding as of 4/27/2022. We filter out sinkable bonds, which brings the total number of CUSIPs to 822,684 observations. Sinkable bonds are removed because there is a lot of “optionality” in the way their prices are calculated. With sinkable bonds, the par owed goes down with each sink, and therefore the math to calculate yield or spread is not straightforward. Sinkable bonds tend to price cheaper due to their structural characteristics, and there is generally a lower buyer pool. We also filter out private placement offerings because such bonds are sold directly to investors via placement agencies and tend to come with specific agreement terms. Additionally, we filter bonds with a settlement period (difference between the sale date and the first settle date) greater than 60 days (approximately 2 months), because long settlement periods require consideration of opportunity cost and because of a lower buyer pool. Finally, we remove CUSIPs without spread data and those with spreads < -500 basis points (bps) or > 500 bps due to likelihood of data error. This brings the total dataset size down to 712,855 observations. For water and sewer revenue bonds only, we filter the larger dataset to only include water and/or sewer revenue bonds, which results in 39,979 CUSIPs for this subset of data.

For the spread at issue analysis, the sample size for both the whole municipal bond market and for water and sewer revenue bonds only is smaller, because the market condition data used to account for differences in the market on each issue date only extends back to early 2015. As such, these two datasets are filtered to only include bonds issued during or after 2015. The brings the total number of CUSIPs down to 623,969 for the whole municipal market, and down to 33,313 for water and sewer revenue bonds only. The latter filter also serves to keep CUSIPs issued during years where we have historical demographic data.

### Data sources

All bond data, including yield and spread data, bond structure, and bond rating, are obtained from Bloomberg (Table 1). Data for each CUSIP are updated nightly. Data for all variables but the market spread were pulled on 5/17/2022, and as such, values for each are current or nearly current. Many of the variables are fixed over time, such as the yield at issue and the bond coupon rate, while the ratings could change, yet the latter is unlikely to occur at a scale that would impact our analysis for the spread at issue.

**Table 1:** General data description.

Data Description	Example Variables	Data Provider	Projected?	Year of Data
Bond data	Price, yield, rating	Bloomberg	No	Current
Climate data	Event-based physical climate risk	Intercontinental Exchange	Yes	Current
Socioeconomic data	Income per capita, percent Black	Intercontinental Exchange	No	Most recent Census year/year of issue
Market condition data	Mutual fund flows, offerings	MMA	No	Current

Climate data and social data are obtained from a third-party data feed from Intercontinental Exchange (ICE) Data Services products (formerly offered by risQ Inc.) that link physical climate risk models and socioeconomic data to each CUSIP, via a novel dataset that links all municipal bonds to their respective geospatial tax-revenue boundaries. The socioeconomic data used are ultimately derived from the American Community Survey (ACS), years 2013–2020. Tract-level demographics are downscaled to a 100m grid using dasymetric modeling, and then aggregated up to an obligor boundary. The physical climate risk scores (“risQ Scores”) are derived via the same modeling approach for each obligor and are based on a weighted combination of insurance-equivalent loss estimates from flood, wildfire, and hurricane catastrophe risk models based on contemporary climate (i.e., climatology linked to today’s global greenhouse gas concentrations). Each integer point increase in the risQ Score can be interpreted as approximately a doubling in financial risk (see Supplement S1 in S1 File for more information). Both the socioeconomic and climate data have been adopted by a growing number of municipal market participants as inputs to a variety of portfolio and risk management decisions [16, 17].

“Market condition” data are provided by Municipal Market Analytics (MMA). MMA provides a daily municipal market index which aggregates variables to reflect the overall market sentiment. Variables included in the index quantify the volume of fund flows, as well as the number of offerings available and the average pricing.

### Spread data and calculation

Municipal bond traders tend to deal in spreads as opposed to yields, where spreads are simply the difference between the yield of the bond and a “risk-free” yield, expressed in basis points (bps) (one bp = 0.01%). For the municipal bond market, tax-exempt bonds trade relative to the Municipal Market Data (MMD) AAA curve, which is a measure of the daily yields of the average tax-exempt AAA municipal bond, for each maturity. There is a daily curve released for maturities from 1–30 years, with the curve generally steepening (increasing) with longer years until maturity. The MMD curve is in part based on the U.S. Treasury curve, and also on specifics of the municipal bond market, such as how well municipal bonds are trading relative to treasuries each day. For taxable municipal bonds, trades are performed relative to the U.S. Treasury curve. For both curves, the spread calculations match the maturity of the municipal bond with that of the curve. A positive spread indicates a lower price than the index bonds and spread generally increases as perceived risk increases and/or as demand decreases. Negative spreads can and do occur if there is high demand for a particular security (often due to tax reasons), driving the prices higher than the AAA index. For example, California bonds often trade through the scale because the scale does not consider the state’s higher tax rates.

Market spreads as of 4/27/2022 are provided directly, through Bloomberg’s BVAL (Bloomberg’s evaluated pricing model), and as such we did not need to calculate spread for those analyses. Market spread values on 4/27/2022 were determined via evaluations, assuming bid-side and 1 million USD and larger block sizes. The municipal market is relatively illiquid, and only ~1% of the market trades on any given day [18]. As such, many of the evaluations in the market spread dataset are estimates, determined through comparable bond sales (much like residential real estate value estimates). The provided market spreads are based on the yield to worst (YTW) of each CUSIP. Spreads at issue are not provided and are therefore calculated using the appropriate MMD and Treasury curves for tax-exempt and taxable municipal bonds, respectively. The MMD and BVAL curves are similar, and thus are comparable for purposes of our study, especially because we use MMD for all spread at issue values and BVAL for all market spread values.

### Dataset characteristics

**Fig 1:**
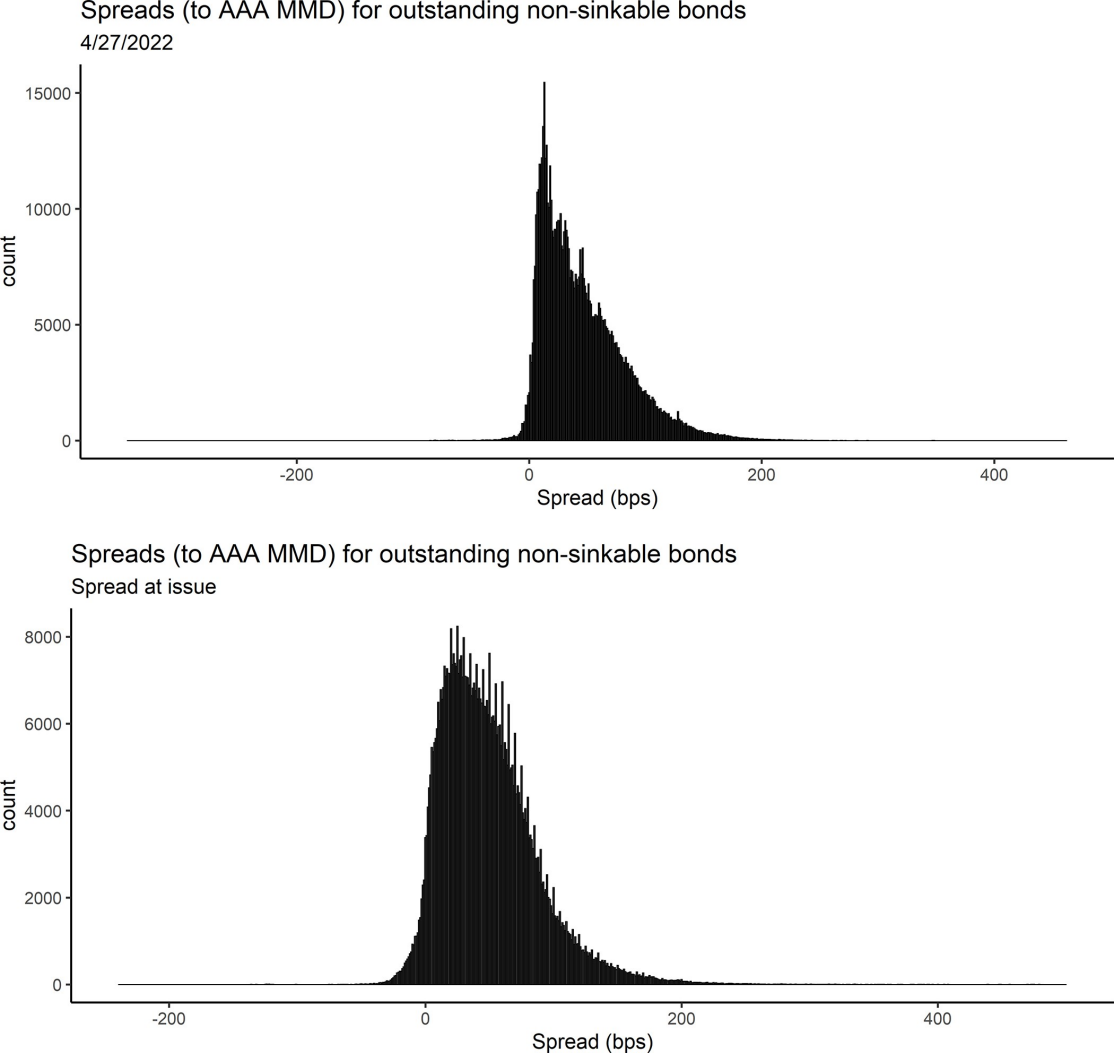
a: Distribution of market spreads, as of 4/27/2022 for all non-sinkable bonds that were not sold via private placement and that do have a settle period > 2 months. 1 bp = 0.01%. b: Distribution of spreads at issue for all non-sinkable bonds issued after 2015 that were not sold via private placement and that do have a settle period > 2 months. 1 bp = 0.01%.

**Fig 2:**
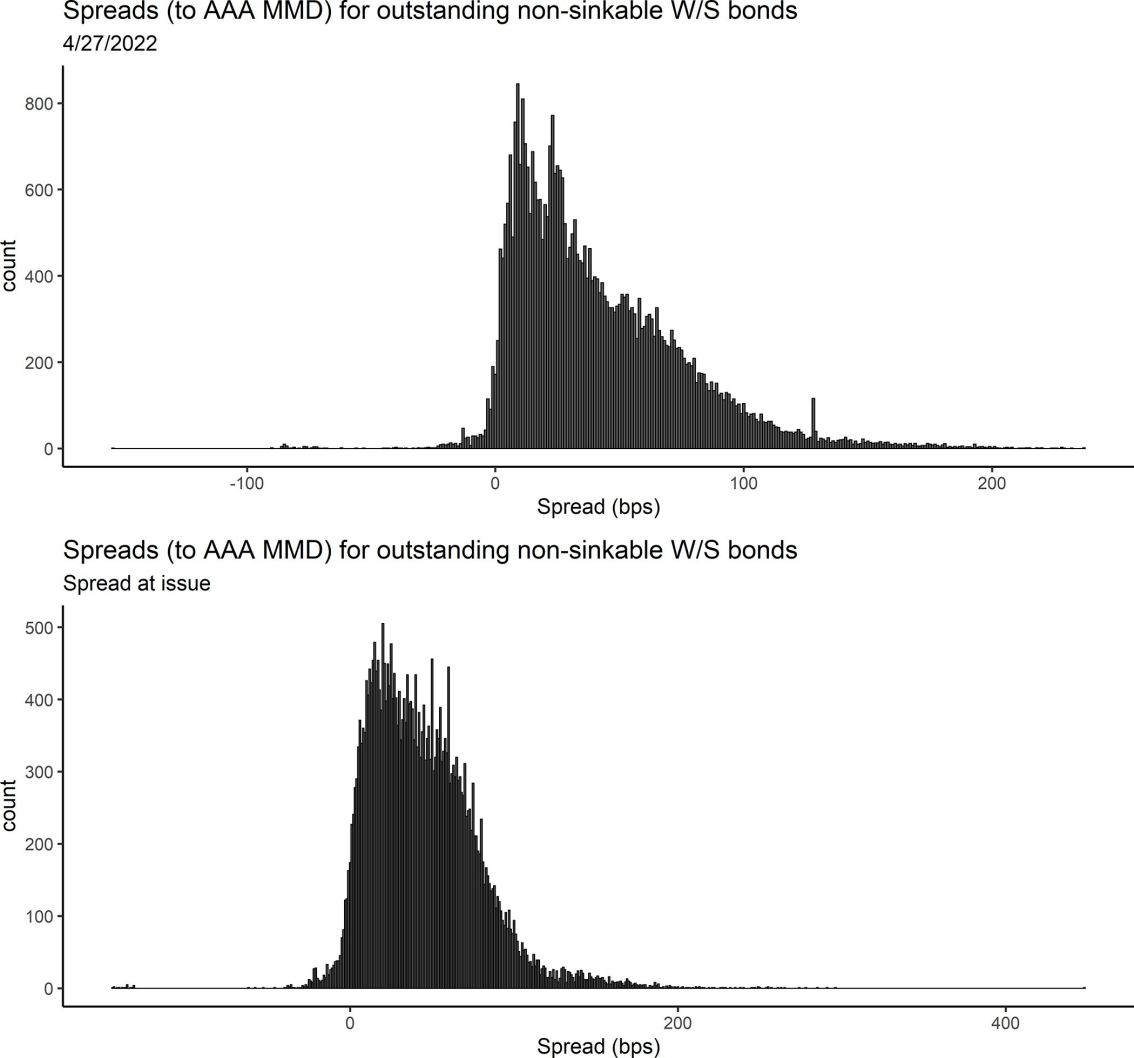
a: Distribution of market spreads, as of 4/27/2022 for all non-sinkable water/sewer revenue bonds that were not sold via private placement and that do have a settle period > 2 months. 1 bp = 0.01%. b: Distribution of spreads at issue for all non-sinkable water/sewer revenue bonds issued after 2015 that were not sold via private placement and that do have a settle period > 2 months. 1 bp = 0.01%.

For the whole market dataset using market spread as the response variable, the average and median spreads are 49.39 and 37.4 bps, respectively. For water and sewer only, the average and median spreads are slightly lower, at 41.14 and 32.6 bps (Table 2). Lower spreads for water and sewer only could be due to lower risk perception of water and sewer revenue bonds relative to other types of municipal bonds. The median number of years until maturity is 6 years for both the whole market and water and sewer only, for market spread as the response variable. For spread at issue as the response variable, the median number of years until maturity is higher, at 10 years. The median ratings are high for all datasets, with a numeric rating median of 2, which equates to AA+. There are some bonds in both datasets with very high and very low spreads (very positive and very negative), yet the vast majority fall between 0 and 100 bps (Figs 1A, 1B, 2A, and 2B).

**Table 2:** Dataset characteristics for the whole market data and water and sewer revenue bonds only, for both response variables: market spread and spread at issue.

	Whole Market			Water and Sewer Only		
	(1)	(2)	(3)	(4)	(5)	(6)
	N	Mean	Median	N	Mean	Median
Market Spread (bps)	712855	49.39	37.4	39979	41.14	32.6
Spread at Issue (bps)	623969	46.04	43	33313	44.38	40
Maturity (years) for market spread dataset	712855	6.65	6	39979	7.02	6
Maturity (years) for spread at issue dataset	623969	10.39	10	33313	10.84	10
Rating for market spread dataset	712855	2.91	2	39979	2.69	2
Rating for spread at issue dataset	623969	2.85	2	33313	2.59	2

**Fig 3:**
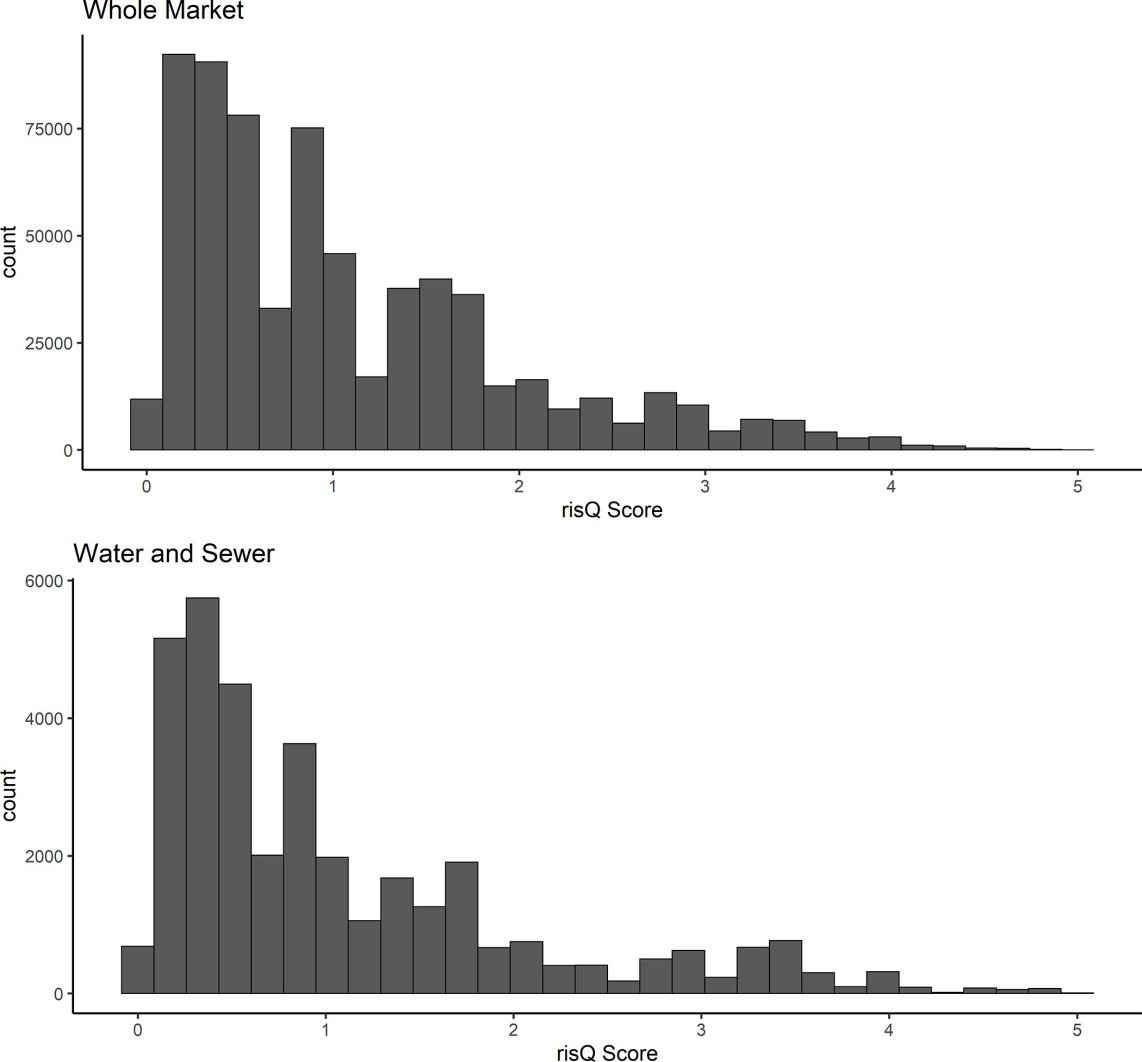
a: Count of bonds outstanding, grouped by climate risk (risQ Score) of the issuer, for the whole market. b: Count of bonds outstanding, grouped by climate risk (risQ Score) of the issuer, for water and sewer revenue bonds only.

**Fig 4:**
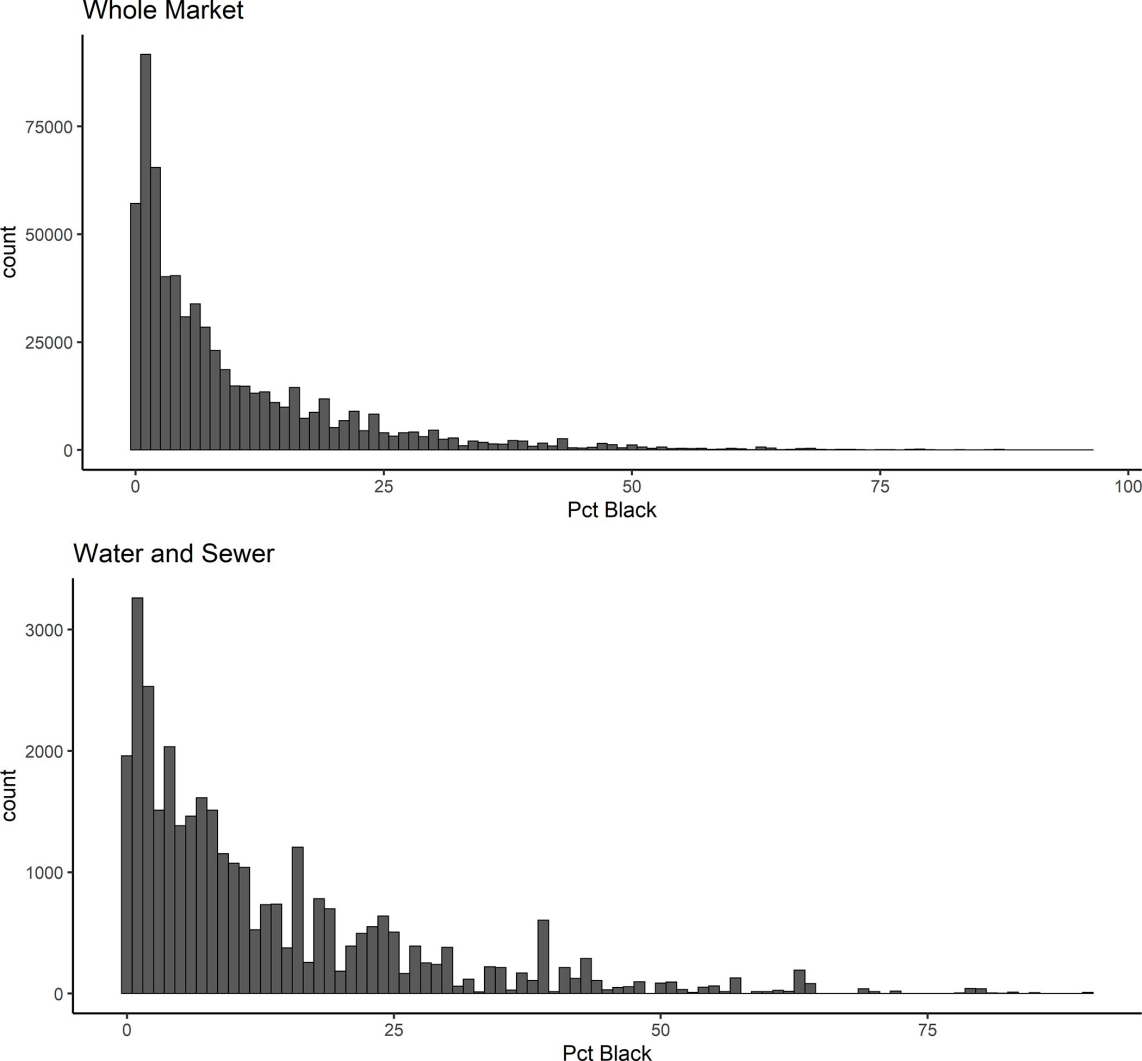
a: Count of bonds outstanding, grouped by percent Black of issuer, for the whole market. b: Count of bonds outstanding, grouped by percent Black of issuer, for water and sewer revenue bonds only.

For each dataset, it is important to recognize potential limitations that may arise due to skewed distributions for both variables ultimately of interest (climate risk and race). For both climate risk and percent Black, most bonds fall towards the left side of the distribution, with a long tail (Figs 3A, 3B, 4A, and 4B). The whole market dataset, due to a much larger sample size, has more bonds from communities with raw risQ Scores of 3–5 and from predominantly (>50%) percent Black communities. While not explored explicitly in this study, many predominantly Black communities are exposed to higher climate risks than other locations in the country. An example of a predominantly Black community with a high risQ Score is Riveria Beach, Florida, which is ~69% Black and has a risQ Score of 4.4.

### Hedonic price model

We first apply a hedonic price model (“base model”) for the whole municipal bond market dataset, and water and sewer only, using both market spread and spread at issue as the response variables. The generalized form for market spread and spread at issue are:

$$Market\;spread=\beta_{1}*Bond\;structure+\varepsilon$$ (1)

$$Spread\;at\;issue=\beta_{1}*Bond\;structure+\beta_{2}*Market\;conditions+\varepsilon$$ (2)

The basic/hedonic model includes (drawing on methods in Bourdeau-Brien and Kryzanowski 2019) [19]:

- The years until the bond matures: “maturity_secondary”/“maturity”
- Issue year, as a factor: “issued_year”
- The coupon %: “CPN”
- The natural log of the par value of the bond at issue (equivalent to the original issue principal), in dollars: “MUNI_ISSUE_SIZE”
- The type of offering, as a factor: “MUNI_OFFERING_TYP”
- The type of coupon, as a factor: “CPN_TYP”
- The security for the bond: “MARKET_ISSUE”—not for water/sewer only because all are revenue bonds
- The market sector of the bond: “MUNI_LONG_INDUSTRY_TYP”—not for water/sewer only because all are water/sewer
- State of issue, as a factor: “STATE_CODE”
- The federal tax status, as a factor, either “Y” or “N”: “MUNI_FED_TAX”—not for water/sewer only because all are tax exempt after applying the filters
- If the bond is insured, as a factor, either “Y”, or “N”: “INSURANCE_STATUS”
- If the bond is callable, as a factor, either “Y”, or “N”: “CALLABLE”
- The offering type or sale type, as a factor: “MUNI_OFFERING_TYP”
- The bond rating, as a category, where A- to AAA = “High IG”, BBB- to BBB+ =“BBB”, and anything lower than BBB- = “Non IG”. Bonds without a rating from either S&P or Moody’s are grouped into the “Unrated” category. While there are other rating agencies (Fitch and Kroll), Moody’s and S&P together rate the vast majority of the municipal bond market. For this analysis, bonds with short term ratings were grouped into the comparable long-term rating category: “rating_cat”

For spread at issue, the base model also includes MMA market conditions data [20]:

- MMA Price Index–a measurement of pricing momentum in the municipal bond market: “MMAPriceIndex”
- MMA Value Index–a qualitative measure of evaluation metrics in the market (i.e., if there is any skew towards overvalued or undervalued): “MMAValueIndex”
- Mutual Fund Flows–a measure of flow into or out of mutual funds, which can be a proxy for weekly demand: “MutualFundFlows”
- Secondary Selling–a measure of bids-wanted in the market: “SecondarySelling”
- Offerings–a measure of par and number of blocks being offered: “Offerings”

### Base model plus non-race socioeconomic data

Owing to the cumulative impacts of historical and/or ongoing racialized disenfranchisement, Black communities and individuals often have lower income and wealth than non-Black places and people [21]. To account for the influence of “economic risk” on the model results, we apply a model that includes non-race socioeconomic factors (non-race SES). The market spread model uses demographic data from the 2020 Census. The spread at issue model uses data from the year of issue. The generalized forms of the base models plus non-race SES are:

$$Market\;spread=\beta_{1}*Bond\;structure+\beta_{2}*Non‐race\;SES+\varepsilon$$ (3)

$$Spread\;at\;issue=\beta_{1}*Bond\;structure+\beta_{2}*Market\;conditions+\beta_{3}*Non‐race\;SES+\varepsilon$$ (4)

The model with these additional economic variables includes the base model variables plus:

- The Gini index of the service area, as a percent, as a measure of income inequality: “Gini_Index”/”gini_indexATISSUE”
- The per capita income of the service area, in dollars: “PerCapInc_Dlrs”/”per_capita_incomeATISSUE”
- The population of the service area, rounded to the nearest thousand: “Population”/”populationATISSUE”

### Base model plus non-race socioeconomic data and climate and race

To examine the influence of physical climate risk and racial bias on municipal credit spreads, we then add the percent Black of the issuer as a variable for race, and the physical climate risk score for the issuer. The market spread model uses race data from the 2020 Census. The spread at issue model uses race data from the year of issue. The generalized forms for the models that include base information, non-race SES variables, and climate and race are:

$$Market\;spread=\;\beta_{1}*Bond\;structure+\;\beta_{2}*\;Non‐race\;SES+\beta_{3}*\;Climate\;risk+\beta_{4}*Race+\;\varepsilon$$ (5)

$$Spread\;at\;issue=\;\beta_{1}*Bond\;structure+\;\beta_{2}*Market\;conditions+\;\beta_{3}*Non‐race\;SES+\beta_{4}*Climate\;risk+\beta_{5}*Race+\;\varepsilon$$ (6)

The model with race and climate variables includes the aforementioned variables plus:

- The risQ Score (0–5 scale), a spatially-relative measure of weighted, insurance-equivalent losses from hurricane, wildfire, and flood risks under current climatology, where every integer point increase suggests an approximate doubling of financial risk; physical climate risk is from the Representative Concentration Pathway 8.5 (the “Business as Usual” International Panel on Climate Change fossil-fuel intensity scenario [22]): “risq_score”
- The percent of the service area that is Black: “PctBlack”

Combined, the models include 24 separate variables (Table 3).

**Table 3:** Model variable definitions.

Model Variable	Definition
maturity_secondary/maturity	The years until the bond matures
issued_year	Issue year of the bond
CPN	The coupon %
MUNI_ISSUE_SIZE	The natural log of the par value of the bond at issue
MUNI_OFFERING_TYP	The type of offering
CPN_TYP	The type of coupon
MARKET_ISSUE	The security for the bond
MUNI_LONG_INDUSTRY_TYP	The market sector of the bond
STATE_CODE	US state of issue
MUNI_FED_TAX	The federal tax status
INSURANCE_STATUS	If the bond is insured
CALLABLE	If the bond is callable
MUNI_OFFERING_TYP	The offering type or sale type
rating_cat	The bond rating, as a category
MMAPriceIndex	A measurement of pricing momentum in the municipal bond market
MMAValueIndex	A qualitative measure of evaluation metrics in the market
MutualFundFlows	A measure of flow into or out of mutual funds
SecondarySelling	A measure of bids-wanted in the market
Offerings	A measure of par and number of blocks being offered
Gini_Index/gini_indexATISSUE	The Gini index of the service area
PerCapInc_Dlrs/per_capita_incomeATISSUE	The per capita income of the service area
Population/populationATISSUE	The population of the service area
risq_score	The climate risQ Score (0–5 scale)
PctBlack	The percent of the service area that is Black

## Results

Overall, all model results show significant and positive coefficients for physical climate risk, yet coefficients per unit risQ Score are too small to suggest any meaningful influence. In contrast, results of regression models largely show that issuers with a higher percent Black are subject to yield penalties, even after accounting for bond structure, rating category, non-race socioeconomic variables (non-race SES) and market conditions (for spread at issue) (Tables 4–7). These results are consistent for both datasets (i.e., whole market and water/sewer only) and both response variables (i.e., market spread and spread at issue), with one exception for the water and sewer only dataset and spread at issue, which shows a non-significant p-value for the percent Black coefficient in the model with climate, race, and non-race SES variables.

**Table 4:** Results for the whole market, using market spread as the response variable. Model 1 is the “hedonic” model, which includes bond structure only. Model 2 includes bond structure and non-race SES. Model 3 includes bond structure, non-race SES, and climate and race.

	Model 1	Model 2	Model 3
maturity_secondary	3.609***	3.803***	3.807***
CPN	-3.459***	-2.732***	-2.766***
MUNI_ISSUE_SIZE	-2.230***	-1.465***	-1.619***
INSURANCE_STATUSY	9.631***	10.693***	10.450***
CALLABLEY	8.949***	9.726***	9.669***
Gini_Index		-28.022***	-32.269***
PerCapInc_Dlrs		0.000***	0.000
Population		0.000	0.000
risq_score			0.841***
PctBlack			0.121***
Num.Obs.	720813	636821	636559
R2	0.498	0.569	0.570
R2 Adj.	0.498	0.569	0.570
RMSE	26.08	23.92	23.88

+ p < 0.1, * p < 0.05, ** p < 0.01, *** p < 0.001

**Table 5:** Results for water and sewer only, using market spread as the response variable. Model 1 is the “hedonic” model, which includes bond structure only. Model 2 includes bond structure and non-race SES. Model 3 includes bond structure, non-race SES, and climate and race.

	Model 1	Model 2	Model 3
maturity_secondary	3.789***	4.220***	4.219***
CPN	-6.114***	-5.614***	-5.619***
MUNI_ISSUE_SIZE	-3.304***	-2.188***	-2.213***
INSURANCE_STATUSY	6.101***	6.314***	6.349***
CALLABLEY	3.460***	2.527***	2.526***
Gini_Index		23.942***	23.372***
PerCapInc_Dlrs		0.000***	0.000***
Population		0.000***	0.000***
risq_score			-0.163
PctBlack			0.011
Num.Obs.	39738	33127	33127
R2	0.535	0.633	0.633
R2 Adj.	0.533	0.632	0.632
RMSE	23.01	20.33	20.33

+ p < 0.1, * p < 0.05, ** p < 0.01, *** p < 0.001

**Table 6:** Results for the whole market, using spread at issue as the response variable. Model 4 is the “hedonic” model, which includes bond structure and market condition. Model 5 includes bond structure, market condition, and non-race SES. Model 6 includes bond structure, market condition, non-race SES, and climate and race.

	Model 4	Model 5	Model 6
maturity	2.673***	2.561***	2.564***
CPN	-2.640***	-3.564***	-3.609***
MUNI_ISSUE_SIZE	-0.842***	-0.004	-0.240***
INSURANCE_STATUSY	17.558***	18.853***	18.405***
CALLABLEY	4.202***	4.320***	4.277***
MMAPriceIndex	0.856***	1.073***	1.043***
MutualFundFlows	-0.255***	-0.277***	-0.276***
SecondarySelling	0.193***	0.151***	0.098*
Offerings	-1.043***	-0.857***	-0.847***
gini_indexATISSUE		-17.936***	-25.073***
per_capita_incomeATISSUE		0.000***	0.000***
populationATISSUE		0.000***	0.000***
risq_score			0.610***
pct_blackATISSUE			0.187***
Num.Obs.	614831	437043	436585
R2	0.557	0.575	0.576
R2 Adj.	0.557	0.575	0.576
RMSE	25.87	25.47	25.39

+ p < 0.1, * p < 0.05, ** p < 0.01, *** p < 0.001

**Table 7:** Results for water and sewer only, using spread at issue as the response variable. Model 4 is the “hedonic” model, which includes bond structure and market condition. Model 5 includes bond structure, market condition, and non-race SES. Model 6 includes bond structure, market condition, non-race SES, and climate and race.

	Model 4	Model 5	Model 6
maturity	2.327***	2.128***	2.129***
CPN	-10.553***	-13.210***	-13.270***
MUNI_ISSUE_SIZE	-1.392***	0.167	-0.030
INSURANCE_STATUSY	12.291***	13.331***	13.702***
CALLABLEY	1.247**	1.723***	1.681**
MMAPriceIndex	2.206***	2.767***	2.793***
MutualFundFlows	-0.690***	-1.248***	-1.248***
SecondarySelling	1.916***	2.007***	2.007***
Offerings	-1.738***	-2.068***	-1.975***
gini_indexATISSUE		5.125	-3.982
per_capita_incomeATISSUE		0.000***	0.000***
populationATISSUE		0.000***	0.000***
risq_score			0.861***
pct_blackATISSUE			0.139***
Num.Obs.	32655	22393	22390
R2	0.508	0.514	0.515
R2 Adj.	0.507	0.512	0.514
RMSE	23.65	23.98	23.94

+ p < 0.1, * p < 0.05, ** p < 0.01, *** p < 0.001

### Market spread results

Market spread results represent the secondary market for municipal bonds. For climate risk, market spreads as of late April 2022 show that a 1-point increase in the risQ Score results in a 0.84 bps increase in yield for the municipal bond market as a whole, significant at the 0.1% level (Table 4). For water and sewer bonds only, however, the coefficient is not significant and is negative in sign (Table 5). This result suggests that physical climate risk is not meaningful for municipal bond prices or evaluations on the secondary market. To put these results in context, the whole market coefficient indicates that a change in risQ Score from 0 (e.g., Liberty County, Kansas) to 5 (e.g., the Florida Keys) would result in an increase in yield of roughly 4.2 bps, which is practically negligible given that this is equivalent to a ~32x increase of insurance-equivalent financial risk from climate.

For race, the market spread results show that a 1 percent increase in the percent Black for an issuer results in a 0.12 bps increase in yield for the municipal bond market as a whole, significant at the 0.1% level (Table 4), and no significant change in yield for water and sewer bonds only (Table 5). The former result means that, holding all else equal, an issuer that is 10% Black issues debt that is 4.8 bps less than an issuer that is 50% Black. This finding is more significant than the climate risk results because a 1% increase in race is a smaller magnitude change than a 1-unit change in risQ Score. For water and sewer bonds only, the market spread results point to little influence of racial bias, yet there are fewer water/sewer bonds issued by higher percent Black communities. Market spread results thus show at least some evidence that the municipal bond market prices in race above and beyond economic risk.

The addition of non-race SES variables (e.g., per capita income) to our base model increases the R-squared from 0.5 to 0.57 for the whole market, and from 0.53 to 0.63 for water and sewer only. The addition of the climate and race does not meaningfully increase R-squared for either model.

### Spread at issue results

Spread at issue results represent the primary market for municipal bonds. For climate risk, yield spreads at issue show that a 1-point increase in the risQ Score results in a 0.61 bps increase in yield for the municipal bond market as a whole (Table 6), and 0.86 bps increase in yield for water and sewer bonds only (Table 7), all significant at the 0.1% level. The spread at issue physical climate risk results indicate that a change in raw risQ Score from 0 to 5 would result in an increase in yield of roughly 3 bps, or 4.3 bps for water and sewer only. The spread at issue results show that water and sewer bonds price in physical climate risk to a greater extent than the whole bond market, which is opposite of the market spread results, yet once again both coefficients are not substantial.

For race, using yield spread at issue, results show that a 1 percent increase in the percent Black for an issuer results in a 0.19 bps increase in yield for the municipal bond market as a whole (Table 6), and 0.14 bps in increase in yield for the water and sewer bonds only (Table 7), both significant at the 0.1% level. Both coefficients are larger than the market spread results. These results mean that, holding all else equal, an increase in the percent of Black individuals in a community by 10% would result in a yield penalty of +1.9 bps for the whole market, and +1.4 bps for water and sewer bonds only. Stated otherwise, a 100% Black community is ascribed a yield penalty of ~19 and ~14 bps, using the whole market and water and sewer model, respectively.

### Caveats

**Fig 5:**
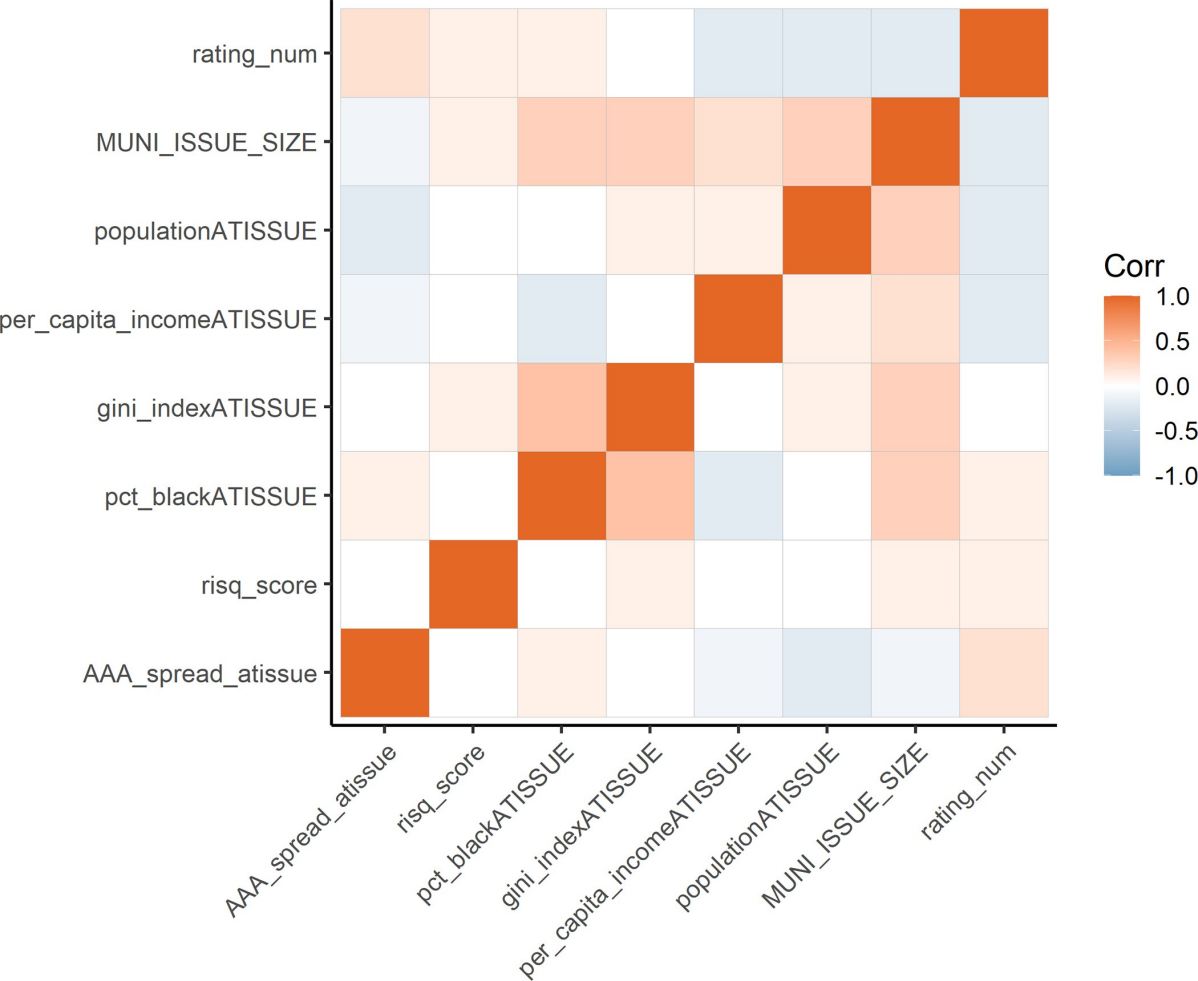
Correlation matrix for continuous variables used in regression models.

There are a few limitations of the hedonic analysis. To begin, use of a linear regression model necessitates consideration of collinearities. Many market variables could induce collinearity: for example, larger issue sizes tend to originate from larger issuers, higher rated bonds tend to have higher incomes, and high climate risk areas tend to have more Black residents (e.g., the Southeast U.S. and Gulf Coast). Race is correlated with all socioeconomic variables added in Models 3 and 6 (Fig 5). Adding variables into the models in a stepwise fashion allows us to parse some inaccurate results that may be arising due to collinearities, yet full accounting of correlations would likely require an alternative approach (e.g., neural networks or decision tree-based models) that can handle complex relationships among predictors while sacrificing ease of model interpretation.

Additionally, the cross-sectional analysis could miss important characteristics of the municipal bond market through time. Ideally, we would evaluate the influence of climate risk and race on municipal credit spreads over time via a longitudinal analysis with panel data. Instead, we take cross sections of spread at two points in time (at issue and on April 27, 2022), which shows the influence of the variables of interest at issue and recently on the secondary market. The secondary market analysis technically shows how the variables influence evaluations, as opposed to actual trades. It is worth noting that 2022 has been a time of high volatility in the municipal bond market (and financial markets generally), and thus the snapshot in time could be different compared to a different point in time (e.g., anytime in 2021). Still, the coefficients generally match in magnitude and direction between the spread at issue and market spread models. Importantly, the cross-sectional analysis does evaluate variable influence at issue, which is most critical from an issuer perspective, as bond pricing at issue affects ultimate cost of capital for issuers.

## Robustness analyses

### Additional climate simulations

We explore the robustness of our climate risk findings via two additional simulations. First, we explore coastal versus non-coastal issuers. Presumably, coastal cities will suffer the most from physical climate risks and rising sea levels [4]. In Supplement S2 in S1 File, we include output from a model for the whole market and spread at issue with a dummy variable for coastal credits (those with sea level rise loss exposure > 0), and an interaction term with years until bond maturity as a way of testing this hypothesis in a continuous manner. We find that non-coastal credits actually have higher coefficients on the risq_score than coastal credits. This is likely due to higher-than-average levels of affluence in coastal communities, which would correlate with lower spreads.

Second, we explore the difference between long-term and short-term bonds, as longer duration bonds would presumably price climate-related risks to a greater degree than shorter-duration bonds [4]. In Supplement S3 in S1 File, we include output from a model for the whole market and spread at issue with a subset of the data. The subset is only bonds that mature in over 10 years. We find that the risq_score coefficient is actually slightly smaller in this analysis than the original that includes all maturities. We take this finding to be more indicative of model insensitivity to the risq_score variable compared to any meaningful difference between the two analyses.

### Clustered standard errors

We test the robustness of all of the coefficients in our analysis via clustering the standard errors. Because of multiple bond issues from the same issuer, where issuers have the same racial and economic variables for each bond, results could overstate significance for every variable in the study, and bias coefficients towards zero [23]. To adjust for this, researchers often invoke the practice of clustering standard errors [24]. We do not find any detectable differences when we follow this practice, where the cluster is defined as the issuer (CUSIP6) (see Supplement S4 in S1 File).

### Randomization test

Due to the collinearities present in the model and the difficulty in fully isolating race from other socioeconomic variables, we also test the robustness of the race coefficient results via a randomization test. We use the entire municipal market dataset and spread at issue as the response variable. We maintain all real data for each CUSIP, except for percent Black, which we randomly shuffle around at the issuer (CUSIP6) level. This random assignment breaks any potential real correlation between race and credit spread. We then run the simulated dataset through a similar model to Model 6 (excluding the climate risk variable) and extract a coefficient for percent Black. We repeat this randomization procedure 1,000 times to obtain a Monte Carlo distribution of coefficients that could be observed by random chance if it were known that there is no correlation between race and credit spread. We then compare the coefficients on race of the Monte Carlo distribution to the true coefficient found in Model 6 (~0.19 bps per 1% increase in percent Black).

**Fig 6:**
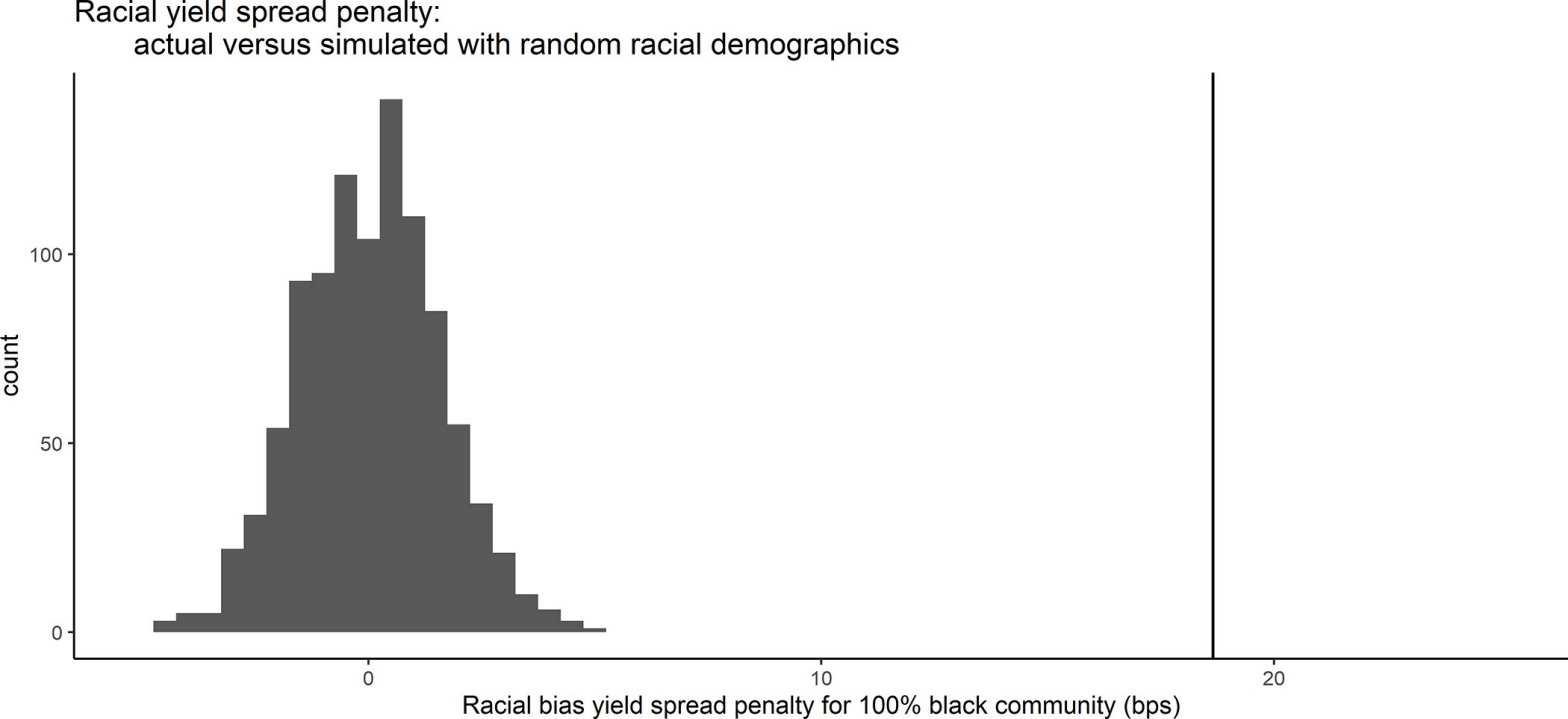
Racial credit spread penalties for a 100% Black community, for 1000 model simulations (histogram) versus actual data (vertical line). The model simulations maintain all real data for each CUSIP, except for race, which is randomly shuffled at the CUSIP6 level.

The results of the simulation corroborate the finding that percent Black is significant and meaningful for municipal credit spreads because none of the Monte Carlo distribution coefficients exceed ~0.05 bps, compared to the true coefficient of 0.19 bps (Fig 6). Said another way, while our actual model results show that a 100% Black community is prescribed a +19 bp penalty on account of race, the simulated results show that if race truly did not matter, and only economic variables moved spreads, then that same community would receive somewhere between a -5 and +5 bps penalty.

## Discussion

The United States relies on the municipal bond market to provide substantial amounts of capital for many of our most basic services, from schools to sewers. The resulting size of the municipal bond market–approximately 4 trillion USD–means that even small systemic trends in yields can have substantial economic impacts nationally, skewing large amounts of capital in important ways. Moreover, because local communities rely on the municipal bond market to finance basic services, and because municipal bonds can represent long-term financial liabilities for a community, any biases in the market that are priced into bond yields can have substantial and long-term implications for the fiscal conditions of a community.

Because of these long-term and financially substantial characteristics of the municipal bond market, there is growing interest in whether the market can appropriately price in climate risk, particularly as the financial implications of climate change become apparent (albeit in somewhat still isolated ways, e.g., wildfires and hurricanes affecting some communities but not most in a state or region). Our results show that while coefficients on the climate variables in our models are slightly positive, physical climate risk is not affecting bond yields at scale. The minimal physical climate risk yield influence, where higher climate risk scores do not result in materially wider spreads, supports a growing body of evidence that the municipal market has yet to factor in environmental risks.

The small coefficients on physical climate risk from our robust analysis of the entire municipal bond universe diverge from the larger coefficients found in other work on climate risk and municipal bond market yields. For example, Painter 2020 found that longer maturity bonds are subject to a 15+ bp penalty for every 1% increase in climate risk [4]. Our model shows that a change in risQ Score of 0 to a risQ Score of 5 results in at most a ~4 bp penalty. For context a risQ Score of 0 means the issuer has the lowest combined hurricane, flood, and wildfire risk compared to all other issuers (e.g., Liberty County, Kansas). A risQ Score of 5 indicates the highest combined hurricane, flood, and wildfire risk compared to all other issuers (e.g., the Florida Keys). That is, in terms of physical climate risk alone, our results suggest that an issuer in the Florida Keys is penalized only 4 bps compared to an issuer in Kansas. Put simply, our results indicate that physical climate risk is not being priced into the municipal bond market. Differences between our results and those of Painter 2020 may be because their study focused exclusively on sea level rise, used county level climate risk estimates, and may have overrepresented large coastal cities.

While climate risk is an emerging area of interest, race has long been known as a factor in U.S. municipal policy, planning, and finance [2, 25]. Yet it is surprising that, to our knowledge, there is no other study that has attempted to look comprehensively at the direct influence of racial composition on yields or spreads in the municipal bond market. Other studies that have analyzed racial bias in the municipal bond have relied on more qualitative approaches; e.g., Ponder 2021 found higher debt issuance costs for predominantly Black cities around the U.S. yet did not statistically separate race from economic risk [26]. Still, the 2021 study points to the likelihood of marginal yield penalties due to racial bias and is advanced as an example of racial capitalism. Dougal et al. 2019 utilize statistical methods but focus on higher education bonds issued by HBCUs, showing that these issuers have higher underwriting fees and wider spreads, even after accounting for standard issuer financials [7]. Most similar to our approach, Bergstresser et al. 2013 found that communities that are more heterogeneous with respect to religion and race receive wider spreads [8]. In building their analysis for fractionalization and its role on municipal credit spreads, they also find that a change in racial composition from 100% white to 100% non-white would result in a +24 bp penalty [8]. Our contribution to this literature is our more direct focus on Black community composition. Our results show a ~19 bp penalty for a change in 100% non-Black to 100% Black.

### Implications

While we show significance for physical climate risk, our coefficients indicate no material yield differential, for either the entire municipal bond market or water and sewer bonds only. This finding partially aligns with expectations, as climate risk data are generally too nascent to be consistently factored into credit metrics. Investors also tend to cite that climate risk has historically not caused many bond defaults, and that the municipal bond market in general has a low default rate. Nevertheless, it can be argued that yield penalties for high climate risk issuers should be much higher, given the dire projections of most modern climate studies and due to the largely underprepared state of U.S. infrastructure with respect to climate resilience [27].

Climate risk is especially relevant for water and sewer issuers, as water and sewer utilities have both infrastructure and services (i.e., the physical water delivery and treatment) that are vulnerable to climate (e.g., changes in precipitation, temperature-induced algal blooms). For all municipal bonds, we would expect a steeper yield curve (one that increases as the maturity of the bond increases) due to uncertainties and projected climate impacts in future decades, and due to mobility of taxpayers and ratepayers in response to climate change. While Painter 2020 finds significance for sea level rise risk for longer duration bonds, our results do not show a difference across maturities. If bond buyers continue to demand more ESG disclosure and initiatives, yield penalties could increase higher than our findings. Until then, however, lower yields despite higher climate risk are an opportunity for issuers to borrow to fund climate resilient infrastructure.

The marginally higher yields for greater percent Black, in contrast, should not exist at all, if the market is truly agnostic about race. Work by historian Destin Jenkins in 2021 was the first major attempt to draw academic attention to the history of the municipal bond market through the lens of structural racism, and how the market may be contributing to ongoing racial inequities [2]. Following 2020, a larger portion of the country, including not only individuals and social justice organizations, but also corporations, major financial institutions, and stakeholders of the municipal bond market, began to more openly grapple with its complex history of slavery, institutional discrimination, and racial capitalism [21, 28]. Still, racial bias in the municipal bond market is not widely considered by investors as material to bond pricing. Professional municipal credit analysts typically avoid injecting race as a variable into their credit work to avoid racial biases. Compliance concerns and business reputation risks further augur against using race to predict bond prices. Our results, however, demonstrate that race is being priced in the municipal bond market, even after controlling for other local socioeconomic factors.

Due to the size of the municipal bond market, with almost 4 trillion in par outstanding, yield penalties attributable to race can have significant financial impacts, nationally, in additional to impacts for individual issuers. The ~19 bp penalty for a 100% Black community relative to a 0% Black community with the same credit and bond structure would result in ~900 million USD in additional interest rate costs for all Black Americans per year (calculated by weighting the total par outstanding by the percent Black for each CUSIP and multiplying by the yield penalty in percentage units). Over a typical duration of a municipal bond (10 years), this equates to 9 billion USD. The exact number is an estimate, yet the order of magnitude dollar impact is significant enough to detract from the ability of higher percentage Black communities to invest in their communities, simply due to higher cost of capital. For climate resilience alone, communities need to maintain and update infrastructure, and higher costs due to racial bias can hinder the efforts of Black communities to manage their climate risk exposure. This is especially important, given that Black communities are disproportionately on the front lines of numerous climate and environmental risks and disasters [29–31].

While our analysis has focused on trends across the entire municipal bond market, it is critical to consider how mispricing of risk in the municipal bond market affects issuers in addition to investors. Our study provides empirical evidence of risk mispricing related to climate and race, and market participants stand to face financial and welfare losses if not corrected. There are numerous reasons that could explain why we find race to be meaningful for municipal bond spreads, including implicit and explicit biases of organizations or individual investors, statistical discrimination, where risk perceptions of decades past have proliferated, and/or circular reasoning on the part of investors who expect others to hold a bias and therefore price accordingly. Regardless of any sort of explanation, race alone should not influence municipal credit spreads. Yet with known real infrastructure, property value, and business interruption risks stemming from climate events, climate risk should matter. From a climate justice perspective, racial bias could further amplify the cost of and reduce capital market access for climate-related and other infrastructure needs for Black communities.

## Supporting information

S1 File (DOCX)
