# Supplementary material for: Climate, race, and the cost of capital in the municipal bond market
Source: PLoS One. 2023 Aug 9;18(8):e0288979. doi: 10.1371/journal.pone.0288979 (PMC10411780; doi:10.1371/journal.pone.0288979)
Supplement: S1 File — (DOCX) [file pone.0288979.s001.docx]

# Supplement S1

## Climate risk (“risQ Score”) methodology

The risQ Score is a 0.0-5.0 score that blends multiple flood, wildfire, and hurricane-related catastrophe models (1, 2) together into one composite relative measure of total insurance-equivalent financial risk to a given municipal debt obligor. An integer increase in the score corresponds to an approximate doubling of financial risk.

Each underlying catastrophe models blends: *hazard* models with economic *exposure* through the medium of *damage/vulnerability* models, all to translate probabilistic risk of climate-related catastrophes to financial losses – *Annual Average Losses (AALs)* -- in a principled, scientific manner (1). There are six catastrophe models that are inputs to the risQ Score (Table S1).

**Table S1: Catastrophe models underlying the risQ Score**

| **Catastrophe Model** | **Category** |
| --- | --- |
| Hurricane Wind | Hurricane |
| Hurricane Storm Surge | Hurricane |
| Hurricane Precipitation-Induced Flood | Hurricane |
| Inland Flood (Non-Hurricane Pluvial and Fluvial) | Flood |
| Coastal Flood (Non-Hurricane) | Flood |
| Wildfire | Wildfire |

AALs are translated into the composite risQ Score system designed to reflect financial risk in a spatially relative manner for any given fixed climatological year. The intention of the risQ Score is to simplify interpretation and enhance usability for stakeholders of the municipal market. The risQ Score weights AALs to reflect the following relativities in total approximate U.S.-aggregate insurance equivalent financial risk over the last several decades: Hurricane: 50%, Flood: 30%, and Wildfire: 20%. These relative contributions are inferred using a combination of historical National Flood Insurance Program (NFIP) claims data and insurance take-up data (e.g., Kousky et al. 2020) to infer historical hurricane and flood losses. A database estimating insurance-equivalent losses from wildfire was also manually constructed with Internet searching to estimate the relative contribution of large wildfires of the past several decades to aggregate financial risk. The distribution of weighted, combined AALs has a distribution with a long right tail. As such, upon translation to a 0.0-5.0 scale, every increase in the risQ Score reflects an approximate doubling of financial risk. The risQ Scores are generally largest for Southeastern U.S. counties owing to hurricane- and coastal flood-related hazards (Figure S1).

Notably, industry research shows that higher risQ Scores correlate to poorer health of the underlying fundamentals of the municipal market, including lower residential property value appreciation, population loss, and mortgage credit risk (4). These insights may be especially worrisome in contrast with our research showing municipal market credit spreads do not yet reflect climate risk.


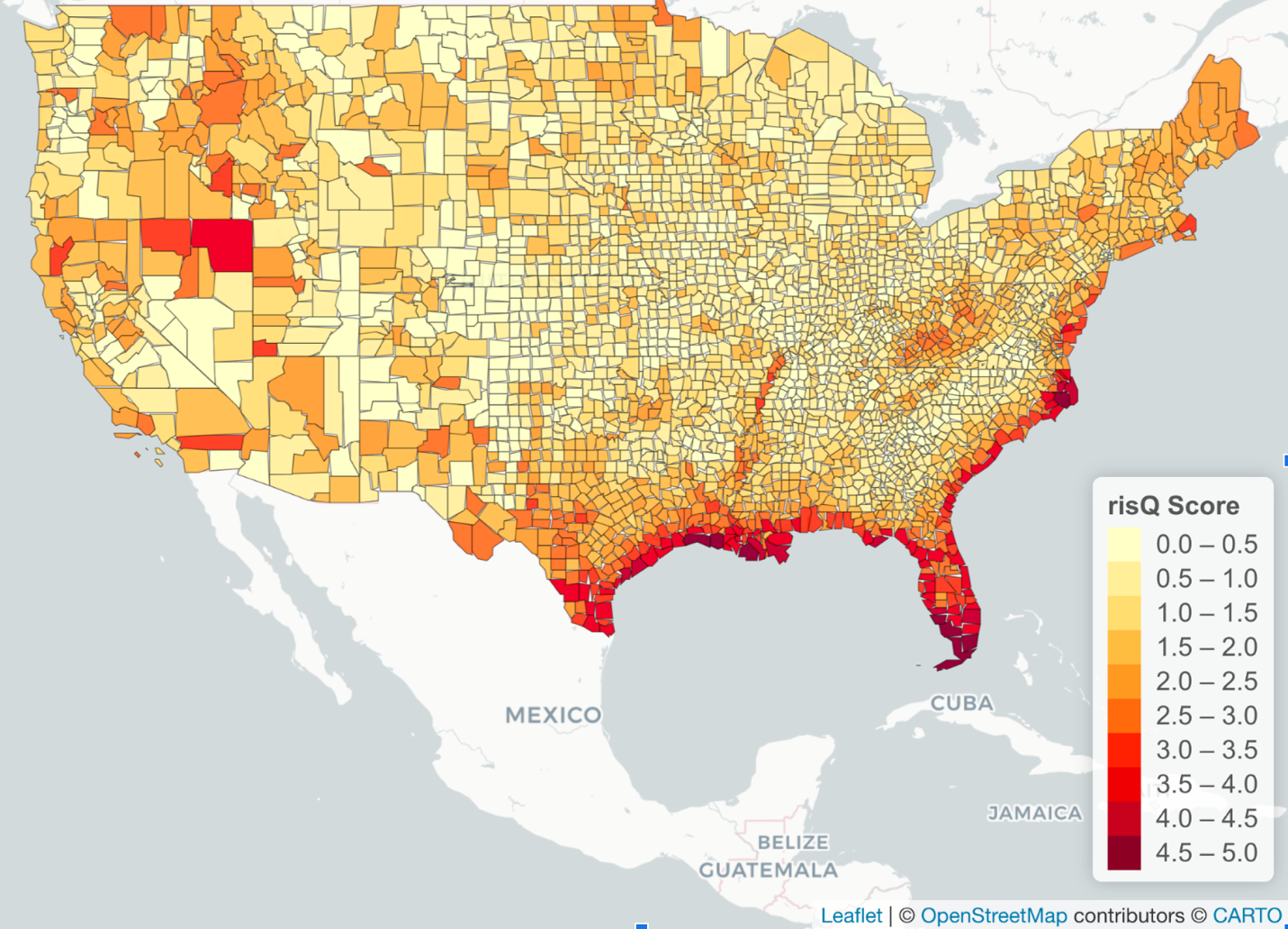


**Figure S1: risQ Score for all counties of the coterminous U.S., for a climatology of 2021 and scenario RCP8.5**

## Supplement S1 References

1. Grossi P, Kunreuther H, Windeler D. An introduction to catastrophe models and insurance. In Catastrophe modeling: A new approach to managing risk 2005 (pp. 23-42). Springer, Boston, MA.
2. Jindrová P, Pacáková V. Natural catastrophe models for insurance risk management. WSEAS Transactions on Business and Economics, volume 16, issue: 1. 2019.
3. Kousky C, Kunreuther H, LaCour-Little M, Wachter S. Flood risk and the US housing market. Journal of Housing Research. 2020 Dec 9;29(sup1):S3-24.
4. Climate risk and U.S. fixed income fundamentals. Intercontinental Exchange [Internet]. 2022 Jun; Available from: https://www.ice.com/insights/climate-risk-and-us-fixed-income-fundamentals#

|  |
| --- |

# Supplement S2

## Longer duration bonds results

**Table S2: Results for modified Model 6 for the whole market, with spread at issue as the response variable, for a subset of the data with bonds maturing in greater than 10 years.**

|  | **Model 6 using Data Subset**  **Bonds Maturing > 10 Years** |
| --- | --- |
| risq_score | 0.309*** |
| pct_blackATISSUE | 0.205*** |
| gini_indexATISSUE | -27.172*** |
| per_capita_incomeATISSUE | 0.000*** |
| populationATISSUE | 0.000*** |
| maturity | 2.121*** |
| CPN | -10.554*** |
| MUNI_ISSUE_SIZE | 1.825*** |
| INSURANCE_STATUSY | 17.611*** |
| CALLABLEY | 6.798*** |
| MMAPriceIndex | 0.861*** |
| MutualFundFlows | -0.480*** |
| SecondarySelling | 0.336*** |
| Offerings | -0.541*** |
| Num.Obs. | 209160 |
| R2 | 0.556 |
| R2 Adj. | 0.556 |
| RMSE | 25.30 |
| + p < 0.1, * p < 0.05, ** p < 0.01, *** p < 0.001 | |

# Supplement S3

## Coastal credits results

**Table S3: Results for modified Model 6 for the whole market, with spread at issue as the response variable, including a dummy variable for coastal credits (sea level rise exposure > 0) and an interaction term with years until bond maturity.**

|  | **Model 6 with Coastal Interaction Term** |
| --- | --- |
| pct_blackATISSUE | 0.187*** |
| gini_indexATISSUE | -24.061*** |
| per_capita_incomeATISSUE | 0.000*** |
| populationATISSUE | 0.000*** |
| maturity | 2.565*** |
| CPN | -3.601*** |
| MUNI_ISSUE_SIZE | -0.211*** |
| INSURANCE_STATUSY | 18.288*** |
| CALLABLEY | 4.256*** |
| MMAPriceIndex | 1.054*** |
| MutualFundFlows | -0.281*** |
| SecondarySelling | 0.102* |
| Offerings | -0.852*** |
| coastalno × risq_score | 1.052*** |
| coastalyes × risq_score | 0.228*** |
| Num.Obs. | 436585 |
| R2 | 0.577 |
| R2 Adj. | 0.576 |
| RMSE | 25.39 |
| + p < 0.1, * p < 0.05, ** p < 0.01, *** p < 0.001 | |

# Supplement S4

## Clustered standard error results

**Table S2a: Results for Model 3 for the whole market, with market spread as the response variable, for both IID and clustered standard errors.**

|  | **Model 3**  **Standard Errors IID** | **Model 3**  **Standard Errors Clustered by CUSIP6** |
| --- | --- | --- |
| risq_score | 0.841*** | 0.841*** |
|  | (0.045) | (0.045) |
| PctBlack | 0.121*** | 0.121*** |
|  | (0.003) | (0.003) |
| Gini_Index | -32.269*** | -32.269*** |
|  | (0.873) | (0.873) |
| PerCapInc_Dlrs | 0.000 | 0.000 |
|  | (0.000) | (0.000) |
| Population | 0.000 | 0.000 |
|  | (0.000) | (0.000) |
| maturity_secondary | 3.807*** | 3.807*** |
|  | (0.009) | (0.009) |
| CPN | -2.766*** | -2.766*** |
|  | (0.035) | (0.035) |
| MUNI_ISSUE_SIZE | -1.619*** | -1.619*** |
|  | (0.029) | (0.029) |
| + p < 0.1, * p < 0.05, ** p < 0.01, *** p < 0.001 | | |

**Table S2b: Results for Model 6 for the whole market, with spread at issue as the response variable, for both IID and clustered standard errors.**

|  | **Model 6**  **Standard Errors IID** | **Model 6**  **Standard Errors Clustered by CUSIP6** |
| --- | --- | --- |
| risq_score | 0.610*** | 0.610*** |
|  | (0.057) | (0.057) |
| pct_blackATISSUE | 0.187*** | 0.187*** |
|  | (0.004) | (0.004) |
| gini_indexATISSUE | -25.073*** | -25.073*** |
|  | (0.920) | (0.920) |
| per_capita_incomeATISSUE | 0.000*** | 0.000*** |
|  | (0.000) | (0.000) |
| populationATISSUE | 0.000*** | 0.000*** |
|  | (0.000) | (0.000) |
| maturity | 2.564*** | 2.564*** |
|  | (0.012) | (0.012) |
| CPN | -3.609*** | -3.609*** |
|  | (0.045) | (0.045) |
| MUNI_ISSUE_SIZE | -0.240*** | -0.240*** |
|  | (0.037) | (0.037) |
| MMAPriceIndex | 1.043*** | 1.043*** |
|  | (0.060) | (0.060) |
| MutualFundFlows | -0.276*** | -0.276*** |
|  | (0.037) | (0.037) |
| SecondarySelling | 0.098* | 0.098* |
|  | (0.040) | (0.040) |
| Offerings | -0.847*** | -0.847*** |
|  | (0.040) | (0.040) |
| + p < 0.1, * p < 0.05, ** p < 0.01, *** p < 0.001 | | |

# Supplement S5

## Results with all variables

**Table S5: Results for Model 6 for the whole market, with spread at issue as the response variable, showing all variables.**

|  | **Model 6** |
| --- | --- |
| risq_score | 0.610*** |
| pct_blackATISSUE | 0.187*** |
| gini_indexATISSUE | -25.073*** |
| per_capita_incomeATISSUE | 0.000*** |
| populationATISSUE | 0.000*** |
| maturity | 2.564*** |
| factor(issued_year)2016 | -1.420*** |
| factor(issued_year)2017 | -3.991*** |
| factor(issued_year)2018 | -5.141*** |
| factor(issued_year)2019 | -2.852*** |
| factor(issued_year)2020 | 5.300*** |
| CPN | -3.609*** |
| MUNI_ISSUE_SIZE | -0.240*** |
| factor(MUNI_OFFERING_TYP)COMPETITIVE | -70.217** |
| factor(MUNI_OFFERING_TYP)LIMITED | 1.252 |
| factor(MUNI_OFFERING_TYP)NEGOTIATED | -61.499* |
| factor(MUNI_OFFERING_TYP)REMARKETED | -59.515* |
| factor(CPN_TYP)ADJUSTABLE, OID | 23.477 |
| factor(CPN_TYP)CASH FLOW | 263.309*** |
| factor(CPN_TYP)FIXED | -4.282+ |
| factor(CPN_TYP)INTER. APPRECIATION | 14.156+ |
| factor(CPN_TYP)INTER. APPRECIATION, OID | 46.308*** |
| factor(CPN_TYP)OID | 5.153 |
| factor(CPN_TYP)TAX CREDIT | 104.446*** |
| factor(CPN_TYP)ZERO COUPON, OID | 51.825*** |
| factor(MARKET_ISSUE)G.O. UNLTD NOTES | -57.669* |
| factor(MARKET_ISSUE)GENERAL OBLIGATION LTD | -12.721*** |
| factor(MARKET_ISSUE)GENERAL OBLIGATION UNLTD | -11.311*** |
| factor(MARKET_ISSUE)REVENUE BONDS | -4.738*** |
| factor(MARKET_ISSUE)SPECIAL ASSESSMENT | 16.600*** |
| factor(MARKET_ISSUE)SPECIAL TAX | -6.470*** |
| factor(MARKET_ISSUE)TAX ALLOCATION | 7.163*** |
| factor(MUNI_LONG_INDUSTRY_TYP)Bond Bank | -11.558*** |
| factor(MUNI_LONG_INDUSTRY_TYP)Development | 13.119*** |
| factor(MUNI_LONG_INDUSTRY_TYP)Education | 0.728 |
| factor(MUNI_LONG_INDUSTRY_TYP)Facilities | -4.305*** |
| factor(MUNI_LONG_INDUSTRY_TYP)General | -4.275*** |
| factor(MUNI_LONG_INDUSTRY_TYP)General Obligation | -6.845*** |
| factor(MUNI_LONG_INDUSTRY_TYP)Higher Education | -6.791*** |
| factor(MUNI_LONG_INDUSTRY_TYP)Housing | 13.813*** |
| factor(MUNI_LONG_INDUSTRY_TYP)Medical | 14.413*** |
| factor(MUNI_LONG_INDUSTRY_TYP)Mello-Roos | 12.887*** |
| factor(MUNI_LONG_INDUSTRY_TYP)Multifamily Hsg | 21.761*** |
| factor(MUNI_LONG_INDUSTRY_TYP)Nursing Homes | 45.171*** |
| factor(MUNI_LONG_INDUSTRY_TYP)Pollution | -5.521*** |
| factor(MUNI_LONG_INDUSTRY_TYP)Power | -13.378*** |
| factor(MUNI_LONG_INDUSTRY_TYP)School District | -11.297*** |
| factor(MUNI_LONG_INDUSTRY_TYP)Single Family Hsg | 14.498*** |
| factor(MUNI_LONG_INDUSTRY_TYP)Student Loan | 75.832*** |
| factor(MUNI_LONG_INDUSTRY_TYP)Tobacco Settlement | 32.209*** |
| factor(MUNI_LONG_INDUSTRY_TYP)Transportation | -7.730*** |
| factor(MUNI_LONG_INDUSTRY_TYP)Utilities | -12.867*** |
| factor(MUNI_LONG_INDUSTRY_TYP)Water | -15.198*** |
| factor(STATE_CODE)AR | 9.081*** |
| factor(STATE_CODE)AZ | 1.085* |
| factor(STATE_CODE)CA | -6.551*** |
| factor(STATE_CODE)CO | -2.616*** |
| factor(STATE_CODE)CT | 0.528 |
| factor(STATE_CODE)DC | -10.601*** |
| factor(STATE_CODE)DE | -8.177*** |
| factor(STATE_CODE)FL | 1.880*** |
| factor(STATE_CODE)GA | -8.736*** |
| factor(STATE_CODE)IA | -4.616*** |
| factor(STATE_CODE)ID | -10.768*** |
| factor(STATE_CODE)IL | 32.504*** |
| factor(STATE_CODE)IN | 3.649*** |
| factor(STATE_CODE)KS | 0.217 |
| factor(STATE_CODE)KY | 15.280*** |
| factor(STATE_CODE)LA | 0.275 |
| factor(STATE_CODE)MA | -4.296*** |
| factor(STATE_CODE)MD | -7.009*** |
| factor(STATE_CODE)ME | -3.534*** |
| factor(STATE_CODE)MI | 12.733*** |
| factor(STATE_CODE)MN | -4.833*** |
| factor(STATE_CODE)MO | -0.670 |
| factor(STATE_CODE)MS | -6.948*** |
| factor(STATE_CODE)MT | -1.367+ |
| factor(STATE_CODE)NC | -9.754*** |
| factor(STATE_CODE)ND | 6.755*** |
| factor(STATE_CODE)NE | -1.637*** |
| factor(STATE_CODE)NH | -2.606*** |
| factor(STATE_CODE)NJ | 7.348*** |
| factor(STATE_CODE)NM | -0.304 |
| factor(STATE_CODE)NV | 12.033*** |
| factor(STATE_CODE)NY | -6.254*** |
| factor(STATE_CODE)OH | -0.002 |
| factor(STATE_CODE)OK | 2.979*** |
| factor(STATE_CODE)OR | -5.005*** |
| factor(STATE_CODE)PA | 11.755*** |
| factor(STATE_CODE)RI | 0.439 |
| factor(STATE_CODE)SC | 0.586 |
| factor(STATE_CODE)SD | -1.481* |
| factor(STATE_CODE)TN | -7.383*** |
| factor(STATE_CODE)TX | 3.833*** |
| factor(STATE_CODE)UT | -6.257*** |
| factor(STATE_CODE)VA | -11.657*** |
| factor(STATE_CODE)VT | 4.792*** |
| factor(STATE_CODE)WA | 1.903*** |
| factor(STATE_CODE)WI | -2.296*** |
| factor(STATE_CODE)WV | 10.354*** |
| factor(STATE_CODE)WY | 2.652 |
| factor(BCA_MUNI_FED_TAX)Y | 48.372*** |
| INSURANCE_STATUSY | 18.405*** |
| CALLABLEY | 4.277*** |
| factor(rating_cat)Non IG | 36.389*** |
| factor(rating_cat)Unrated | 49.023*** |
| MMAPriceIndex | 1.043*** |
| factor(MMAValueIndex)issuer | 1.889*** |
| factor(MMAValueIndex)neutral | 1.014*** |
| MutualFundFlows | -0.276*** |
| SecondarySelling | 0.098* |
| Offerings | -0.847*** |
| Num.Obs. | 436585 |
| R2 | 0.576 |
| R2 Adj. | 0.576 |
| RMSE | 25.39 |
| + p < 0.1, * p < 0.05, ** p < 0.01, *** p < 0.001 | |
